# The Effects of *In Vitro* Incubation of Asthenoteratozoospermic Semen after Density Gradient Centrifugation at Room Temperature and 37°*C* on Sperm Parameters, Chromatin Quality and DNA Fragmentation in a Short Time Period

**DOI:** 10.18502/jri.v21i4.4332

**Published:** 2020

**Authors:** Motahareh Karimi Zarchi, Behnam Maleki, Mahmood Dehghani Ashkezari, Leila Motamed Zadeh, Azam Agha-Rahimi

**Affiliations:** 1- Department of Biology, Medical Biotechnology Research Center, Ashkezar Branch, Islamic Azad University, Yazd, Iran; 2- Research and Clinical Center for Infertility, Yazd Reproductive Sciences Institute, Shahid Sadoughi University of Medical Sciences, Yazd, Iran

**Keywords:** Asthenoteratozoospermia, DNA fragmentation, *In vitro* incubation, Room temperature

## Abstract

**Background:**

Sperm quality is an important factor in assisted reproductive technology (ART) that affects the success rate of infertile couples treatment. *In vitro* incubation of sperm can influence its parameters and DNA integrity. The present study focused on the effect of different incubation temperatures sperm parameters on asthenoteratozoospermia semen prepared with density gradient centrifugation at different times.

**Methods:**

Twenty-seven samples were collected and prepared. Then, the suspension was divided into two parts. One part was incubated at room temperature (RT), and another was incubated at 37°*C*. Immediately and after 2 *hr* (2H) and 4 *hr* (4H), spermatozoa were evaluated regarding motility, viability, morphology, sperm protamine deficiency, chromatin and DNA fragmentation. Statistical analysis was performed using paired t-test and repeated measures. The p<0.05 was considered statistically significant.

**Results:**

Our results showed that following 2 and 4 *hr* of incubation at RT, sperm progressive motility and viability decreased significantly. Sperm DNA fragmentation increased significantly following 2 and 4 *hr* of incubation at RT and 37°*C*. The Trend analysis confirmed that there were no significant differences between sperm parameters and DNA fragmentation after different times at RT and 37°*C*.

**Conclusion:**

Incubation of sperm at RT in comparison to 37°*C* didn’t preserve sperm parameters and DNA efficiently. Therefore, IVF, ICSI and IUI procedure should be performed in the soonest possible time after sperm preparation.

## Introduction

Sperm quality is an important factor in assisted reproductive technology (ART) that affects the success rate of infertile couples treatment (1). Men with total motile sperm count <40% and, or progressive motility less than 32% are classified as asthenozoospermic (2). Intracytoplasmic Sperm Injection (ICSI) can overcome suboptimal motility by injecting one sperm into the ooplasm. Today, many ICSI cycles in the ART laboratory is performed on asthenozoospermic samples. Density gradient centrifugation (DGC) is the preferred sperm preparation technique for asthenozoospermic samples.

In delayed oocyte pickup cycle, prepared sperm is incubated 2–4 *hr in vitro* before ICSI. The temperature during *in vitro* incubation of prepared sperm and duration of it can influence the quality of the sperm (3–8). Avoiding an iatrogenic damage to the prepared sperm is an important issue. However, in most textbooks and manuals, there is no recommendation about time and temperature for sperm *in vitro* incubation after sperm preparation (2, 9). Bourne and Archer recommended incubation temperature of 37*°C* under 5% CO_2_ (IVF) or room atmosphere (ICSI) (10).

*In vitro* incubation of ejaculated spermatozoa at 37*ºC* has been recommended for liquefaction and widely used in ART and andrology laboratories (2). After semen processing, the spermatozoa are routinely kept at 37*°C* until using for IVF. It was previously demonstrated that long-term *in vitro* incubation at 37*ºC* reduced the motility, viability, and increased sperm DNA fragmentation (4–8). There are several studies about the adverse effect of incubation time on sperm parameters. It has been shown that incubation of density gradient prepared human spermatozoa at 37°*C* was associated with significant loss of motility in normozoospermia (6, 11). Also, it is reported that prolonged incubation of normal human semen after preparation will increase sperm DNA fragmentation (7). Although these studies confirmed the adverse effect of incubation time on sperm quality, they are not focused on incubation temperature in a short time period.

The main objective of this study was evaluation of the effect of *in vitro* incubation of prepared sperm at 37*°C* and room temperature that is routinely practiced in andrology and ART laboratories on sperm parameters and chromatin status in asthenoteratozoospermic ‎men. The rational in selecting the patients was that most ICSI treated cases were asthenoteratozoospermic.

## Methods

### Patients:

In this study, twenty-seven asthenoteratozoospermic samples were collected from men undergoing infertility treatment between July 2018 and March 2018. Only samples with sperm motility (Progressive+Non-progressive) below 32% and morphology below 4% were included in this study. Exclusion criteria were the men with the age more than 40 years, smoking, alcohol consumption and varicocele. The samples were obtained from patients who referred to Yazd Research and Clinical Center for Infertility. All the patients were signed the informed consent form. Author’s institute review board approved this study (Ethics code: IR. IAU.YAZD.REC.1398.018).

### Study design:

Semen samples were obtained by masturbation. The abstinence period of participants was between 3 and 7 days. Semen analysis was carried out according to WHO guidelines. Sperm motility was reported as the percentage of progressive, non-progressive, and immotile spermatozoa. Sperm viability was evaluated by Eosin-Nigrosin staining test. Papanicolaou staining was used for morphology assessment (2).

**Figure 1.**
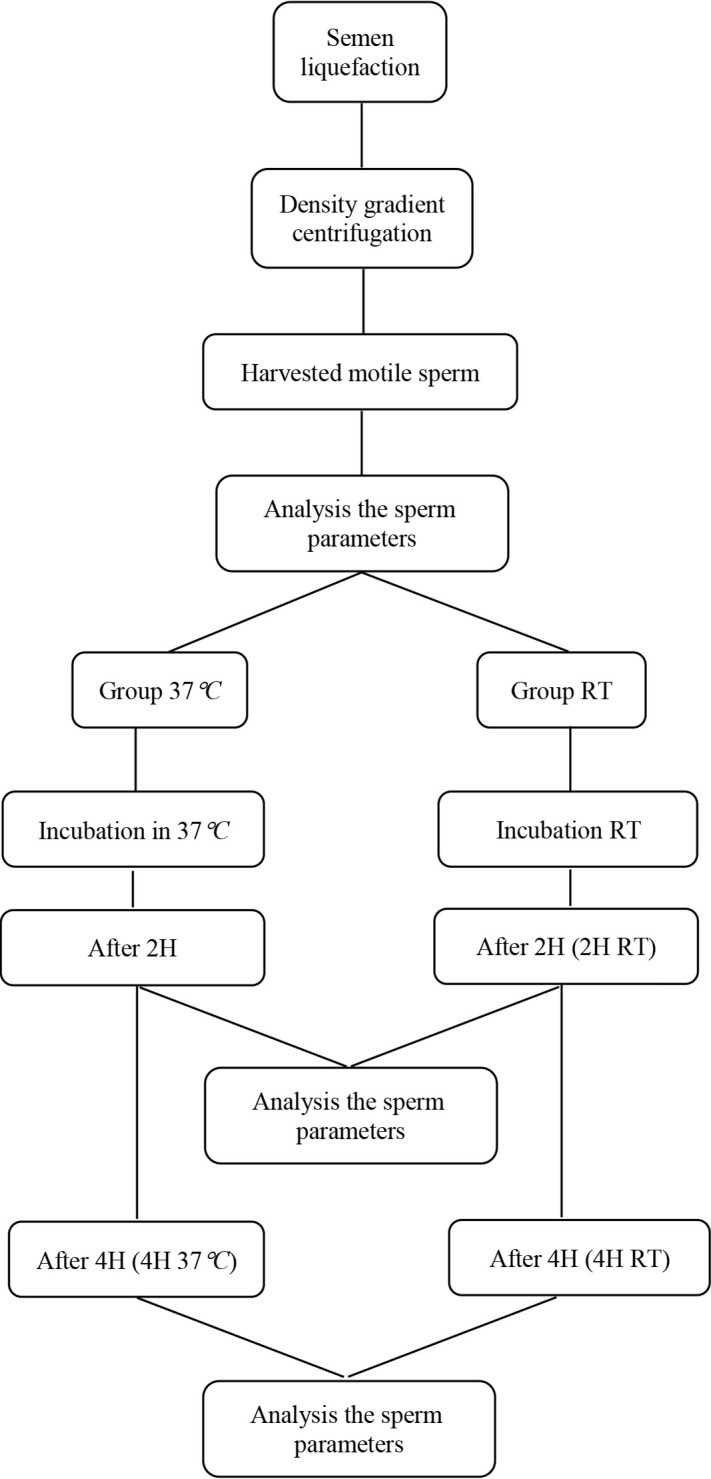
The schematic diagram of the experiment design. RT= room temperature

Semen preparation was done by density gradient centrifugation. After initial evaluation, the retrieved suspension was divided into two parts. One part was incubated at room temperature (RT), and another was incubated at 37°*C*. After 2 *hr* (2H) and 4 *hr* (4H), spermatozoa were evaluated regarding motility, viability, morphology, sperm protamine deficiency, chromatin, and DNA fragmentation in both groups (Figure 1).

### Density gradient centrifugation (DGC):

A two-layer gradient (80% and 40%) was prepared by diluting SpermGrad™ (Vitrolife Inc., Sweden). Next, 1 *ml* of liquefied semen sample was placed on top of the upper layer into a 15-*ml* conical Falcon tube (12).

The tube was centrifuged at 300–400 g for 15–30 min. The supernatant was discarded, and the pellet was washed twice with 5 ml of Hamsf10 supplemented with HSA (5 *mg/ml*) followed by centrifugation at 200 *g* for 5 *min*. The supernatant was discarded, and the pellet was re-suspended in a volume of 0.5 *ml* of Hamsf10 supplemented with HSA (5 *mg/ml*). Following gradient centrifugation, samples were divided into two aliquots and incubated for 2 *hr* and 4 *hr* at either 1) room temperature (23–24*°C*) and 2) 37*°C* humidified incubator.

### Assessment of sperm chromatin, DNA integrity and DNA fragmentation:

The air-dried smears were fixed in a solution of 3% glutaraldehyde in 0.2 *M* phosphate buffer, (14 *ml* of 10.2 *M* NaH_2_PO_4_ plus 36 *ml* of 0.2 *M* Na_2_HPO_4_, pH=7.2) for 30 *min*. Fixed smears were stained with the solution of 5% AB in 4% acetic acid (pH=3.5) for 5 *min* and then rinsed with distilled water (13). Stained (Abnormal) and unstained (Normal) spermatozoa were counted under a light microscopy with ×1000 magnification. The results present the percentage of stained spermatozoa.

The air-dried smears were fixed in a solution of ethanol-acetone (1:1) at 4°*C* for 30 *min*. The hydrolysis of smears was performed by HCl (0.1 molar) for 5 *min*. Then, TB dye solution (0.05% TB in 50% McIlvaine's citrate phosphate buffer at pH=3.5) was used for 10 *min*. Finally, the slides were rinsed in distilled water and dehydrated with ethanol and xylene at room temperature for 3 *min* (14). The stain of pale blue was considered as normal, and dark blue or violet/purple as abnormal. Abnormal spermatozoa (TB+) were reported as percentage.

### Sperm chromatin dispersion (SCD) assay:

The SCD test was according to the SCD kit (SDFA, Idea Varzan Farad Co., Iran) protocol. Aliquots of low melting-point agarose in microtubes are present in the kit. The Eppendorf tube was placed in a water bath at 100*°C* until the agarose melted completely, and then in a water bath at 37*°C*. Next, 50 *μl* of the diluted semen sample was added to each tube, and mixed gently. Then, a 50 *µl* of the mixture was loaded on a pre-coated slide (Provided in the kit) and covered with a coverslip and placed on a cold plate for 5 *min*. The coverslip was gently removed, and the slide was embedded in solution A in darkroom. Each slide was immersed in solutions B for 30 *min*. After washing with distilled water, the samples were dehydrated for 2 *min* in increasing ethanol concentration solution (70%, 90%, and 100%) for dehydration and left to dry at RT. Finally, each slide was embedded in solution C for 75 *s*, solution D for 3 *min* and solution E for 2 *min*. The slide was washed in distilled water and allowed to dry. The assessment was performed under the ×1000 magnification and light microscopy (15). The halo size around sperm heads was determined in comparison with the core width of spermatozoa. The small halo showed high DNA fragmentation, and the large ones showed DNA integrity. The result presents the percentage of spermatozoa with high DNA fragmentation.

### Statistical analysis:

Statistical analysis was performed using paired t-test. The p<0.05 was considered significant. Also repeated measure analysis was done to measure the changes that occur over time for groups. If Test of Sphericity was significant, the p-value of Greenhouse-Geisser was read for Trend analysis. If Test of Sphericity was not significant, the p-value of Sphericity Assumed was read for Trend analysis. The graphs for Trend analysis were exported from SPSS. Data were represented as box and whisker plots, whereby boxes depict the 25th and 75th percentiles with the indication of the median value, and whiskers depict the 10th and 90th percentiles. These graphs were exported from Prism software and data is reported as mean±SD.

## Results

### Effect of incubation time and temperature on sperm parameters:

**Figure 2.**
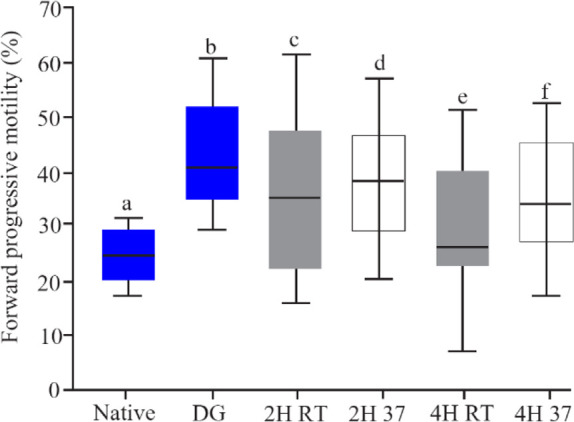
The rate of forward progressive motility in native semen, after DG preparation and *in vitro* culture, boxes depict the 25th and 75th percentiles with indication of the median, and whiskers depict the 10th and 90th percentiles, a, b (p< 0.0001), b, c (p=0.0.001), b, d (p=0.02), b, e (p<0.001), b, f (p=0.001) c, d (p=0.5), e, f (p=0.1)

The effect of temperature and incubation time on sperm motility is presented in figure 2. A significant increase in the progressive motility (41.77±13.31) rate and total motility (Progressive+non-progressive) rate of spermatozoa was observed after density gradient sperm preparation. The progressive motility rate decreased significantly after 2 *hr* incubation at RT (34.19±16.54, p=0.001) and 37*ºC* (36.46±13.53, p=0.01). This decrease was significant after 4 *hr* in both groups. The progressive motility was not different between RT and 37*ºC* after both 2 and 4 *hr* incubation.

**Figure 3.**
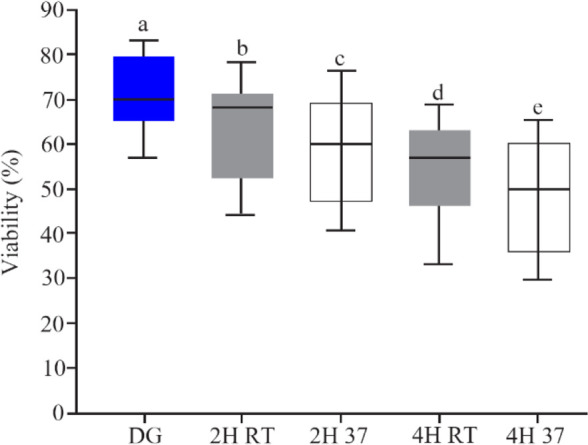
The rate of sperm viability after DG preparation and *in vitro* culture, boxes depict the 25th and 75th percentiles with indication of the median, and whiskers depict the 10th and 90th percentiles, a, b (p<0.001), a, c (p<0.001), a, d (p<0.001), a, e (p<0.001), b, c (p=0.3), d, e (p=0.07)

Figure 3 shows the effect of temperature and incubation time on sperm viability. For the samples incubated at RT, viability decreased significantly after 2 *hr* (63.18±12.91, p<0.001), and 4 *hr* (53.88±11.68, p<0.001); also at 37*ºC*, a significant decrease occurred after both 2 *hr* (59.51± 12.99, p<0.001) and 4 *hr* (47.11±15.47, p<0.001) in comparison to initial time (70.96±11.04). The viability rate was not different between RT and 37*ºC* after both 2 and 4 *hr* incubation.

The results of sperm morphology analysis in both the pre-incubated and post-incubated groups are illustrated in table 1. The proportion of morphologically normal spermatozoa was not significantly different after 2 and 4 *hr* in both incubated temperatures (RT and 37*ºC*) compared with DGC prepared group.

**Table 1.** The rate of normal morphology, chromatin condensation, DNA structure and packaging status of spermatozoa in the groups

**Variable**	**DG**	**2H RT**	**4H RT**	**2H 37**	**4H 37**
**Normal morphology**					
	2.2±0.9 (1.8–2.5)	2.2±1.0 (1.8–2.6)	2.1±0.9 (1.8–2.5)	2.0±0.8 (1.7–2.4)	2.0±0.8 (1.7–2.4)
**Aniline blue**					
	52.7±15.4 (46.5–59.0)	52.1±15.0 (46.1–58.0)	52.3±15.0 (46.4–58.3)	50.8±14.7 (45.0–56.7)	52.8±14.0 (47.1–58.5)
**Toluidine blue**					
	76.8±10.7 (72.2–81.3)	75.6±10.5 (71.4–79.8)	73.3±15.8 (67.0–79.6)	73.9±16.1 (67.4–80.5)	73.0±16.2 (66.6–79.4)

The data presented as mean±SD (95% CI). DGC=Density Gradient Centrifugation. No significant difference was found between groups

### Sperm chromatin and DNA fragmentation:

**Figure 4.**
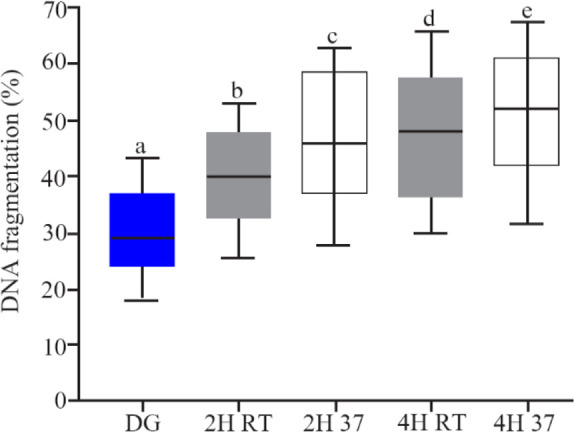
The rate of sperm DNA fragmentation after DG preparation and *in vitro* culture, boxes depict the 25th and 75th percentiles with indication of the median, and whiskers depict the 10th and 90th percentiles, a, b (p<0.001), a, c (p< 0.001), a, d (p<0.001), a, e (p<0.001), b, c (p=0.049), d, e (p= 0.3)

Table 1 shows the data on sperm chromatin condensation, DNA structure and, packaging status. There were not statistically significant differences between different groups. DNA fragmentation in different groups is shown in figure 4. DNA fragmentation increased significantly (p<0.001), followed by *in vitro* culture of spermatozoa after 2 and 4 *hr* at both RT and 37*ºC*. There was no statistically significant difference in DNA fragmentation between RT and 37*ºC* after 4 *hr*, although this index was lower borderline values at RT after 2 *hr* than 37*ºC* (p=0.049).

### Further comparison of temperature effects:

Repeated measures analysis was used in this study to measure changes occurred for samples over time. Trend analysis showed all of the factors evaluated in this study were not significant between the two groups (Figure 4).

## Discussion

After semen processing, the spermatozoa were routinely kept in 37*°C* incubator until use. There are several purposes for *in vitro* incubation of spermatozoa. Preservation of sperm could be used for delayed oocyte pickup, *in vitro* maturation of immature metaphase I oocytes, rescue ICSI in the following day, *etc*. However, there is no determined agreement about the condition of *in vitro* incubation of sperm prior to use in ART.

The purpose of this study was to investigate the changes in sperm quality parameters after semen preparation and *in vitro* sperm incubation at RT and 37°*C* in asthenoteratospermia. There are several studies about the effect of incubation time on sperm parameters, but they are not focused on incubation temperature in a short time. Only Thijssen et al. evaluated the effect of temperature on sperm quality parameters, but their study was about the long term incubation (6).

The results of this study showed a significant loss of sperm motility, when processed sperm was incubated at RT or 37°*C*. This was in line with results reported by Schuffner et al. who showed incubation of density gradient prepared human spermatozoa at 37°*C* was associated with significant loss of motility in normozoospermic men (11). Furthermore, Thijssen et al. reported a significant decline in the motility of spermatozoa in both DG and swim-up samples after incubation at RT and 37°*C* after 24 *hr*, although RT preserves better motility (6).

It is proposed that, when spermatozoa are incubated at lower temperatures, they adopt an inactive state, which allows them to preserve their energy. Accordingly, when spermatozoa enter the female reproductive tract and face high temperatures, they would become hyperactive (16). This hypothesis possibly explains the reduction in motility of spermatozoa at 37°*C* compared with lower temperatures (6, 17). In the present study, decrease of motility was observed after a short incubation at both RT and 37°*C*, and there was no significant difference between 37°*C* and RT. The same results were observed about viability. The decrease in sperm viability rate may be associated with a decrease in glucose concentration and accumulation of products such as pyruvate followed by *in vitro* culture of sperm. Another possible explanation for viability loss is the production of ROS. The accumulation of ROS was also associated with damage to cell and organelle membrane which led to loss of viability (18).

In this study, the change in morphological characteristics has not been observed. Contrary to our result, Thijssen et al. reported that the proportion of morphologically normal spermatozoa was significantly decreased after *in vitro* incubation in both DG and swim up samples. However, the incubation time in their study was 24 *hr* (6). In addition, Peer et al. reported that *in vitro* incubation of sperm samples for 2 *hr* at 37°*C* caused significant decrease in the morphologic integrity of the sperm nuclei compared with the initial state. However, no significant morphologic changes in sperm nuclei were observed after incubation at 21*°C*. They concluded that prolonged sperm incubation in assisted reproduction techniques should be performed at 21*°C* rather than 37*°C*. They used the motile sperm organelle morphology examination (MSOME) technique for morphology evaluation (19).

Abnormalities in sperm chromatin have been extensively considered as a source of male infertility in recent years, and several chromatin specific staining methods have been introduced for male infertility diagnosis in past decades (20–24). In this context, chromatin condensation, DNA structure, and packaging status of spermatozoa were evaluated with acidic aniline blue and toluidine blue staining methods, respectively. Our result showed there were no significant increase in positive predictive value of aniline blue and toluidine blue after *in vitro* incubation.

Recently, the DNA fragmentation assay has been widely used in male infertility diagnosis. Our study showed *in vitro* incubation of DG prepared sperm at both RT and 37°*C* causes an increase in DNA fragmentation. Nabi et al. demonstrated higher rates of sperm DNA fragmentation following the incubation of prepared normozoospermic samples at 37*°C* (7). Matsuura et al. concluded DNA fragmentation increased after incubation both at room temperature (RT) and at 37°*C* in air, but room temperature incubation causes less DNA fragmentation after 24 *hr*. This study was about raw semen without preparation (17). In another article, it was reported that in long time incubation (24 *hr*), RT better preserves sperm quality (6). Our study showed prepared asthenoteratospermia semen sample has high DNA fragmented nuclei. DNA fragmentation index was reported higher in men with asthenoteratozoospermia in comparison to fertile ones (25, 26). Our results reveal that short *in vitro* incubation of these sperm cause an increase in DNA fragmentation in both groups. Although the paired t-test analysis showed that DNA fragmentation after 2 *hr* was moderate at RT, but further analysis including Trend analysis showed the incubation at RT does not prevent sperm damage.

**Figure 5.**
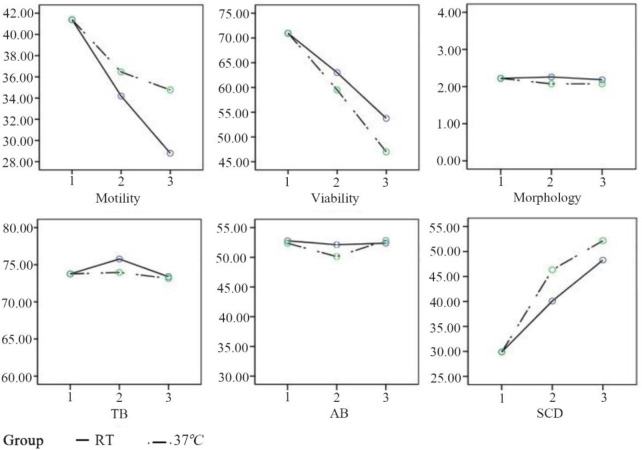
The comparison of sperm parameters with trend analysis. The results show the parameters were similar in all experiments. Y= Estimated Marginal Means. TB=Toluidine blue, AB=Aniline blue. The graphs exported from SPSS software

Trend analysis is used when changes occur over time for different samples. Figure 5 shows the results of this analysis. These results confirmed that all parameters changed similarly over time in two different conditions including RT and 37°*C*. This reveals that incubation of sperm at RT in comparison to 37°*C* didn’t preserve sperm parameters and DNA efficiently.

Accordingly, *in vitro* incubation of spermatozoa cells for 2 or 4 *hr* at both RT and 37°*C* has adverse effect on sperm parameters such as motility and viability and DNA fragmentation. When using conventional IVF, ICSI, IMSI or, IVM, it is a routine practice to prepare the sperm before microinjection and keep it for 2 *hr* at 37°*C* incubator or, RT which may have an adverse effect on overall clinical outcome. In this regard, Pujol et al. recently reported that clinical pregnancy rates diminish progressively when the time between oocyte pick up and ICSI is increased. They demonstrated that each 1 *hr* increase in the oocyte pick up -ICSI time reduced the likelihood of biochemical pregnancy by 7.3% and of clinical pregnancy by 7.7% (27).

## Conclusion

In conclusion, the results of this study show that a short *in vitro* incubation (2 or 4 *hr*) at both RT and 37°*C* has a detrimental effect on DNA fragmentation and sperm parameters of prepared asthenozospermic semen sample. Hence, the findings of this study recommend IVF, ICSI and IUI should be done in the soonest possible time in order to minimize the diminishing quality of the prepared sperm.
